# Biodegradable Chitosan-*graft*-Poly(l-lactide) Copolymers For Bone Tissue Engineering

**DOI:** 10.3390/polym12020316

**Published:** 2020-02-04

**Authors:** Maria Kaliva, Anthie Georgopoulou, Dimitrios A. Dragatogiannis, Costas A. Charitidis, Maria Chatzinikolaidou, Maria Vamvakaki

**Affiliations:** 1 Institute of Electronic Structure and Laser, Foundation for Research and Technology-Hellas (FORTH-IESL), 70013 Heraklion, Greece; mchatzin@materials.uoc.gr (M.C.); vamvakak@iesl.forth.gr (M.V.); 2 Department of Materials Science and Technology, University of Crete, 70013 Heraklion, Greece; anthieg87@yahoo.com; 3 Research Unit of Advanced, Composite, Nano Materials & Nanotechnology, School of Chemical Engineering, National Technical University of Athens, 9 Heroon Polytechniou St., Zographou, 15780 Athens, Greece; ddragato@chemeng.ntua.gr (D.A.D.); charitidis@chemeng.ntua.gr (C.A.C.)

**Keywords:** chitosan, poly(l-lactide), graft copolymers, CS-*g*-PLLA, bone tissue engineering, pre-osteoblastic cells, MC3T3-E1

## Abstract

The design and synthesis of new biomaterials with adjustable physicochemical and biological properties for tissue engineering applications have attracted great interest. In this work, chitosan-*graft*-poly(l-lactide) (CS-*g*-PLLA) copolymers were prepared by chemically binding poly(l-lactide) (PLLA) chains along chitosan (CS) via the “*grafting to*” approach to obtain hybrid biomaterials that present enhanced mechanical stability, due to the presence of PLLA, and high bioactivity, conferred by CS. Two graft copolymers were prepared, CS-*g*-PLLA(80/20) and CS-*g*-PLLA(50/50), containing 82 wt % and 55 wt % CS, respectively. Degradation studies of compressed discs of the copolymers showed that the degradation rate increased with the CS content of the copolymer. Nanomechanical studies in the dry state indicated that the copolymer with the higher CS content had larger Young modulus, reduced modulus and hardness values, whereas the moduli and hardness decreased rapidly following immersion of the copolymer discs in alpha-MEM cell culture medium for 24 h. Finally, the bioactivity of the hybrid copolymers was evaluated in the adhesion and growth of MC3T3-E1 pre-osteoblastic cells. In vitro studies showed that MC3T3-E1 cells exhibited strong adhesion on both CS-*g*-PLLA graft copolymer films from the first day in cell culture, whereas the copolymer with the higher PLLA content, CS-*g*-PLLA(50/50), supported higher cell growth.

## 1. Introduction

Despite the intrinsic ability of small bone defects to heal spontaneously with minimal cure, the treatment of large bone defects, resulting from trauma or disease, still remains a big challenge in orthopedics, with limited success until recently, due to the complicated structure of bone [1,2,3]. Tissue engineering has emerged as a promising therapeutic approach for the restoration of the function of damaged or diseased skeletal tissue. It is based on a multi-component system that combines cells, bioactive molecules and porous three-dimensional (3D) scaffolds, composed of biodegradable biomaterials, aiming to stimulate the inherent ability of bone to regenerate by promoting osteogenesis, osteoconductivity and osseοintegration [2,4,5].

An important factor that determines the success of bone tissue engineering is the design and synthesis of biomaterials that can provide an ideal micro-environment and architecture for tissue development. Biomaterials for use in tissue engineering should be biocompatible and bioactive, as well as present controllable biodegradability and appropriate mechanical properties, and they should be able to form structural scaffolds and promote regenerative processes via the effective delivery of bioactive cues [1,2,5]. A wide range of biomaterials have been employed to fabricate scaffolds for bone tissue regeneration. Among them, chitosan (CS), a natural, cationic polysaccharide, has been extensively used due to its biocompatibility, bioactivity, biodegradability, low immune response, antimicrobial and wound healing properties, and its structural similarities with the glycosaminoglycans (GAGs), the major component of the bone extracellular matrix (ECM), which plays an important role in cell–cell adhesion [6,7,8,9,10]. Studies have shown that CS demonstrates significant osteoconductive properties: it promotes cell adhesion and the proliferation of osteoblast and mesenchymal cell lines, and stimulates neovascularization, enhancing the formation of functional bone in vivo [6,8,11]. However, the use of CS in tissue engineering is limited by its poor mechanical properties and insolubility in neutral water. To improve its processability and physicochemical properties, CS has been combined with synthetic polymers [12,13]. Among them, a promising candidate to ameliorate CS’s properties is polylactide (PLA), a chiral polymer which exists in the L and D stereoisomers, poly(l-lactide) (PLLA), poly(d-lactide) (PDLA), respectively. PLA is a thermoplastic polyester synthesized using monomers derived from renewable resources, and has been extensively used in bone tissue engineering, due to its biocompatibility, biodegradability, non-toxicity, good processability and excellent mechanical properties. Moreover, PLA has been approved by Food and Drug Administration (FDA) for clinical use [14,15].

The combination of chitosan and PLA can lead to novel multi-functional biomaterials with improved properties, suitable for biomedical applications. The most facile method to obtain such composites is by simply mixing the two polymers, however, the homogeneous mixing of CS with PLA is challenging, due to their dissimilar structure and properties. Melt processing is hindered by the high melting temperature of CS, higher than its decomposition temperature, whereas solution processing suffers from the absence of a common good solvent for the two polymers (CS dissolves only in highly acidic aqueous media, whereas PLA is soluble only in organic solvents) [16].

To improve their miscibility, CS/PLA nanofibers and porous scaffolds were developed by electrospinning and lyophilization, respectively, in the presence of a nonionic surfactant [15,17]. Other studies combined the two polymers by depositing or chemically binding CS on the surface of PLA membranes [18], films [19], scaffolds [20] and fibers [21]. However, in all these cases, the deposition of CS on the surface of PLA resulted in the early removal of the polysaccharide from the surface during degradation. 

Another feasible and very promising approach to combine the two polymers involves the chemical grafting of PLA chains along the CS backbone. Two approaches have been employed so far to bind PLA onto CS, the “*grafting from*” and the “*grafting to”* techniques [22]. In the “*grafting to”* method, pre-synthesized end-functional PLA chains are chemically bound onto CS via its amino or hydroxyl functionalities [23], whereas, in the “*grafting from*” method, the ring-opening polymerization of the lactide monomer is carried out from the CS functional groups [24,25]. Amphiphilic CS-*g*-PLLA copolymers have been used as drug carriers [26,27,28,29], however, only very few studies have employed CS-*g*-PLLA copolymers in tissue engineering to develop fibrous [25] or porous scaffolds [30]. Cytotoxicity experiments on electrospun CS-*g*-PLLA fibers using fibroblast cells showed that the graft copolymers with the higher LLA content present high cell viability, higher than CS alone, and comparable to that of pure PLLA [25], while mechanical testing of the samples showed that the CS-*g*-PLA fibrous scaffolds exhibit improved tensile strength and modulus, rendering them promising for tissue engineering applications [31]. Furthermore, the in vitro and in vivo biocompatibility of CS-*g*-PLLA porous scaffolds has been evaluated using bone marrow mesenchymal stem cells (BMSCs), verifying the excellent biocompatibility and histocompatibility of the graft copolymers compared to the individual precursor materials [30]. In another study, the blood coagulation and biocompatibility of CS-*g*-PLLA copolymers was confirmed using HUVEC and L929 cells, rendering them promising candidates for biomedical applications [32]. 

In the present work, we combined the good mechanical and biological properties of PLLA with the biocompatibility and bioactivity of CS, in CS-*g*-PLLA graft copolymers and investigated, for the first-time, the biological response of MC3T3-E1 pre-osteoblast cells on these materials. Copolymers of two different compositions (55 wt % and 82 wt % CS) were prepared via the “*grafting to*” method. First, PLLA chains were synthesized via the ring-opening polymerization of LLA, using an organic amine to avoid the toxic metal catalysts, and were then chemically grafted onto the CS backbone via amine coupling chemistry. The graft copolymers were characterized by ^1^H Nuclear Magnetic Resonance (^1^H NMR) and Fourier-transform infrared (FTIR) spectroscopies, thermogravimetric analysis (TGA), differential scanning calorimetry (DSC) and X-ray diffraction (XRD) measurements. The nanomechanical properties and the degradation profiles of the copolymers were also examined using CS-*g*-PLLA-compressed discs. Finally, the potential of the CS-*g*-PLLA copolymers in bone tissue engineering was investigated by examining the ability of CS-*g*-PLLA films to support the adhesion, growth and proliferation of MC3T3-E1 pre-osteoblast cells in vitro.

## 2. Materials and Methods

### 2.1. Materials

CS (low molecular weight (50–190 KDa), degree of deacetylation, DD ~ 85% calculated by ^1^H NMR spectroscopy), N,N-dimethyl-4-aminopyridine (99%, DMAP), glycolic acid (GA), sodium dodecyl sulfate (SDS), N,N’-Dicyclohexylcarbodiimide (DCC) and N-hydroxysuccinimide (NHS) were obtained from Sigma (St. Louis, MO, USA). 3,6-Dimethyl-1,4-dioxane-2,5-dione (l-lactide, LLA), purchased from Alfa Aesar (Thermofisher, Kandel, GmbH Germany), was recrystallized from ethyl acetate three times, and dried under vacuum prior to use. Tetrahydrofuran (THF) was dried by refluxing in the presence of potassium under an inert atmosphere. Phosphate buffer saline (PBS) and paraformaldehyde were purchased from Sigma (St. Louis, MO, USA). PrestoBlue^®^ reagent for cell viability and proliferation, minimum essential Eagle’s medium (α-MEM), trypsin/EDTA, penicillin/streptomycin and fetal bovine serum (FBS) were purchased from Molecular Probes by Life Technologies, (ThermoFisher Scientific Carlsbad, CA, USA). All other chemicals and solvents were of analytical grade and were used without further purification.

### 2.2. Synthesis of Carboxyl Terminated PLLA

PLLA with a terminal carboxylic acid group (PLLA-COOH) was synthesized by ring-opening polymerization using an organic catalyst (DMAP) and glycolic acid as the initiator. In a typical synthesis, LLA (8.00 g 55.5 mmol), glycolic acid (0.090 g, 1.18 mmol) and DMAP (0.135 g, 1.10 mmol) were charged in a round bottom flask. Subsequently, the flask was sealed with a rubber septum, degassed by three freeze–vacuum–thaw cycles, and placed in a thermostated oil bath at 100 °C for 6 h. The obtained solid was dissolved in chloroform, precipitated in methanol and dried under vacuum [33].

### 2.3. Synthesis of CS-g-PLLA Copolymers

For the preparation of the CS-*g*-PLLA copolymers, the pre-synthesized PLLA-COOH chains were grafted onto the CS backbone, following a procedure described earlier [34,35]. Two CS-*g*-PLLA copolymers were prepared, CS-*g*-PLLA(80/20) and CS-*g*-PLLA(50/50), by varying the % weight ratio of CS/PLLA-COOH in the reaction feed from 80/20 to 50/50 *w*/*w* %, respectively. In a typical synthesis for the preparation of CS-*g*-PLLA(80/20), a 2 wt % aqueous solution of CS (1.0 g, repeat unit 6.0 mmol) containing 2 *v*/*v* % acetic acid was prepared and was mixed with 20 mL of -aqueous SDS (1.1 g, 3.8 mmol) solution. The mixture was kept overnight at room temperature to allow the SDS/CS complex (SCC) to form. The precipitate was isolated by filtration, washed extensively with water and freeze-dried. Next, the activated ester of PLLA-COOH was prepared by dissolving PLLA-COOH (0.25 g, 0.029 mmol), NHS (0.0067 g, 0.058 mmol) and DCC (0.012 g, 0.0,058 mmol) in 10 mL dry THF. The above solution was stirred at room temperature under a N_2_ atmosphere for 24 h, and then filtered and dried under vacuum, overnight. The obtained product was dissolved in N-methylpyrrolidinone and was subsequently added to 100 mL solution of SCC in dimethyl sulfoxide (DMSO) under continuous stirring. The reaction was allowed to proceed at 40 °C for 48 h under a N_2_ atmosphere. Finally, the non-grafted PLLA-COOH chains were eliminated by dialysis (MW cut-off 14,000) against DMSO, while the SDS was removed from the SCC-*g*-PLLA graft copolymer by precipitation of the product in a 15 wt % tris(hydroxymethyl)aminomethane (Tris) aqueous buffer solution. The precipitate was washed several times with the Tris solution and water to remove the SDS and next with DMF to eliminate any unreacted PLLA-COOH chains. Finally, the copolymers were washed extensively with water to remove the DMF traces and then lyophilized to obtain a foam product. For the synthesis of CS-*g*-PLLA(50/50) the same procedure as described above was followed. In particular, 1.0 g of CS (repeat unit 6.0 mmol) was used to be modified with SDS while 1.0 g of PLLA-COOH (0.12 mmol) was activated by using 0.028 g of NHS (0.24 mmol) and 0.049 g of DCC (0.24 mmol).

### 2.4. Characterization Techniques

Attenuated total reflectance (ATR)-FTIR spectra were recorded on a Nicolet 6700 optical spectrometer (Thermo Scientific, Waltham, MA, USA). For each spectrum, 128 scans were collected in the range of 400–4000 cm^−1^. ^1^H NMR spectra were measured on a Bruker AMX-500 spectrometer (Bruker, Rheinstetten, Germany). The molecular weight and molecular weight distribution of PLLA-COOH were determined by gel-permeation chromatography (GPC). The instrument was equipped with a Waters 515 HPLC pump (Waters, Milford, MA, USA), two PL mixed-D and mixed-E columns and a Waters 410 refractive index detector (Waters, Milford, MA, USA) operated at 35 °C. Calibration was based on a series of six narrow MW linear poly(methyl methacrylate) standards, ranging from 850 to 342,900 g·mol^−1^, and THF was used as the eluent at a flow rate of 1 mL·min^−1^. XRD measurements were performed to examine the crystallinity of PLLA-COOH, CS and the CS-*g*-PLLA copolymers. XRD patterns were obtained on a PANanalytical X’pert Pro MPD powder diffractometer (PANanalytical, Lelyweg, The Netherlands) (40 kV, 45 mA) using CuKa radiation (λ = 1.5418°). The thermal stability of CS-*g*-PLLA, PLLA-COOH and CS was studied by TGA using a Perkin Elmer Pyris Diamond TG/DTA instrument (Perkin Elmer, Llantrisant, UK). In a typical measurement, 10–15 mg of the sample were placed in a platinum holder and were heated under a constant nitrogen flow from room temperature up to 500 °C at a heating rate of 10 °C/min. Finally, the thermal transitions of CS, PLLA-COOH and CS-*g*-PLLA were investigated using a Polymer Laboratories 12,000 DSC (Polymer Laboratories, Church Stretton, UK). All measurements were carried out under a nitrogen flow and the temperature was varied between 20 and 200 °C at a heating rate of 10 °C/min. In the first heating run, CS and the CS-*g*-PLLA copolymers were heated at 100 °C for 20 min to remove any chemically bound water, followed by an increase in temperature to 200 °C.

### 2.5. Preparation and Characterization of the CS-g-PLLA Copolymer Films and Discs

CS-*g*-PLLA(80/20) and CS-*g*-PLLA(50/50) films were deposited onto glass substrates by spin-coating using a SPS spin-coater (model Spin 150, SPS-Europe, Putten, The Netherlands). A total of 160 μL of a 1 *w*/*v* % copolymer solution in H_2_O/CF_3_COOH (50 *v*/*v* %) were spun at 2000 rpm for 160 sec. The films were dried under vacuum at 60 °C for 24 h before being neutralized by rinsing with a 0.1 M NaOH solution for several minutes, then washed with water and dried under a N_2_ flow. 

For the preparation of the compressed discs, approximately 80 mg of PLLA-COOH, CS-*g*-PLLA(80/20) or CS-*g*-PLLA(50/50) were pressed using a SPECAC mechanical compacting press (SPECAC-LTD, Orpington, UK). A pressure of 15 tons was applied for 5 min to prepare the discoid samples. The surface morphology of the CS-*g*-PLLA films was examined by atomic force microscopy (AFM) on a multimode Nanoscope III instrument (Digital Instruments, Veeco, Santa Barbara, CA, USA) operated at a tapping mode at 1 Hz scan rate and by field-emission scanning electron microscopy (SEM, JEOL 7000, JEOL, Tokyo, Japan). The wettability of thin polymer films was examined by water contact angle measurements using a surface tensiometer (DataPhysics OCA-40, DataPhysics Instruments GmbH, Filderstadt, Germany). The contact angles were determined by depositing a 5 µL droplet of water onto the surface of the films. Each measurement was carried out in triplicate (n = 3). 

### 2.6. Nanoindentation Testing in the Dry and Wet State

Nanoindentation tests were performed with a Hysitron TriboLab^®^ nanomechanical test instrument using a two-dimensional force displacement transducer (Hysitron TriboLab^®^test instrument, Minneapolis, MN, USA). The transducer can apply loads in the range of 1–10,000 μN with a resolution of 1 nN, while the maximum penetration depth recorded is 3000 nm (3 μm) with a resolution of 0.04 nm. A Berkovich indenter (Hysitron TriboLab^®^test instrument, Minneapolis, MN, USA) with a 100 nm tip radius was used for the measurement of the hardness (H), elastic modulus (E) and reduced modulus (Er) values of the CS-*g*-PLLA copolymers. Experiments were performed in a clean area environment with ~45% humidity and 23 °C ambient temperature [36]. H and E values were extracted from the experimental data (unload displacement curves) using the Oliver and Pharr method [37,38], in which the derived expressions are based on Sneddon’s elastic contact theory [39]. Tests at 50–1000 μN with different hold times (5, 10 and 20 s) at the maximum applied load were performed in order to investigate the time dependent and viscoelastic behavior of the synthesized materials. The depth profile experiments followed trapezoidal loading–unloading curves, depending on the hold time. For the measurements in the wet state, both copolymers were immersed in α-MEM at 25 °C and their nanomechanical behavior was measured after certain time periods (1, 2, 3, 6 and 10 days, 3 and 4 weeks). Samples immersed in α-MEM exhibited lower resistance to applied load, and stronger adhesion between the tip and the sample’s surface, and thus the nanoindentation tests were conducted following the displacement control mode at 500 nm displacement depth with holding times 5 and 10 s for CS-*g*-PLLA(50/50) and CS-*g*-PLLA(80/20), respectively, and an identical 10 s loading and unloading time. All H and E values presented and calculated in this study are an average (±STDV) of three measurements.

### 2.7. Degradation Study

For the in vitro degradation study, CS-*g*-PLLA discs were placed in 10 mL PBS (pH 7.4) and were incubated at 37 °C. Every seven days, the samples were removed from the medium, freeze-dried and the weight loss was calculated using the following formula:

D = (W_o_ − W_1_)/W_o_ (1)

where, W_o_ is the initial weight of the dried discs and W_1_ is their weight at the certain time point of the measurement. 

### 2.8. Cell Culture of MC3T3-E1 pre-Osteoblastic Cells on the CS-g-PLLA Films

MC3T3-E1 pre-osteoblastic cells were isolated from newborn mouse calvaria. Cells were obtained from DSMZ GmbH (Germany; DSMZ no: ACC 210) and have the capacity to differentiate into osteoblasts and osteocytes in vitro. Pre-osteoblastic cells were cultured in α-ΜΕΜ medium supplemented with 10% fetal bovine serum (FBS) and 1% penicillin/streptomycin. Once a week, when the cells reached confluence, they were passaged using trypsin/EDTA. Experiments were carried out using cells between Passage 8 and 15. When the cells reached ~ 90% confluence in the flasks, they were subcultured following detachment with 0.25% Trypsin/EDTA and resuspension in culture medium. A total of 6 × 10^4^ MC3T3-E1 cells were seeded onto the sterilized CS-*g*-PLLA films. The films were placed in 24-well Corning^®^ plates, and tissue culture polystyrene (TCPS) was used as a control substrate.

### 2.9. Adhesion and Morphology of the MC3T3-E1 Cells on the CS-g-PLLA Films

The morphology of the pre-osteoblastic cells on the CS-*g*-PLLA films was monitored using SEM (JEOL JSM-6390 LV, JEOL, Tokyo, Japan) after 2 days in culture. At this timepoint, the cells were rinsed twice with PBS buffer, fixed with 2 *v*/*v* % para-formaldehyde and 2 *v*/*v* % glutaraldehyde for 15 min at room temperature and dehydrated in increasing concentrations (30–100 *v*/*v* %) of ethanol. The films were dried in a critical point dryer (Baltec CPD 030, BAL-TEC AG, Balzers, Liechtenstein, Switzerland), sputter-coated with a 20 nm thick layer of gold (Baltec SCD 050, BAL-TEC AG, Balzers, Liechtenstein, Switzerland) and observed at an accelerating voltage of 15 kV.

### 2.10. Viability and Proliferation of the Pre-Osteoblastic Cells on the CS-g-PLLA Films

A total of 6 × 10^4^ pre-osteoblastic cells were used for the viability and proliferation assays. The metabolic activity of the cells on the CS-*g*-PLLA films was assessed with the PrestoBlue^®^ assay (Invitrogen, Carlsbad, CA, USA). When cells are viable, the nontoxic metabolic indicator resazurin is reduced to a red product resorufin, which can be detected photometrically. At each timepoint of 1, 4 and 7 days in culture, 400 μL of 1:10 diluted PrestoBlue^®^ reagent in primary culture medium α-MEM were added directly to each well and were incubated at 37  °C for 30 min. The supernatants of the samples were transferred to another 24-well plate and the absorbance at 570 and 600 nm was measured by means of a spectrophotometer (Synergy HTX Multi-Mode Microplate Reader, BioTek, Winooski, VT, USA). For each experiment, three replicates were used (n = 3).

### 2.11. Statistical Analysis

Statistical analysis was performed using ANOVA (GraphPad Prism 5 software) in order to evaluate the differences between the CS-*g*-PLLA films as well as the reference material, TCPS. Experimental results are presented as mean values ± standard error of the mean. A p < 0.05 was considered significant. 

## 3. Results

### 3.1. Synthesis of Carboxyl Terminated Poly(L-Lactide)

**Scheme 1:**
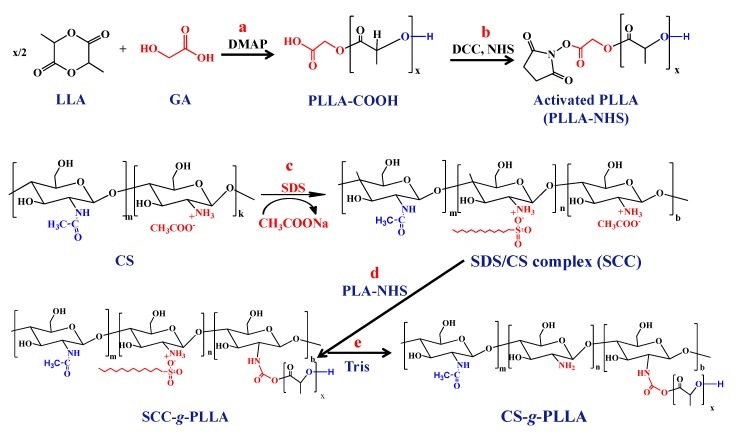
Schematic representation of the synthetic procedure followed for the preparation of the CS-*g*-PLLA copolymers: (**a**) ring-opening polymerization of LLA; (**b**) activation of PLLA-COOH; (**c**) formation of the organo-soluble SCC complex; (**d**) grafting of PLLA-NHS onto the SCC backbone; (**e**) elimination of SDS.

**Figure 1:**
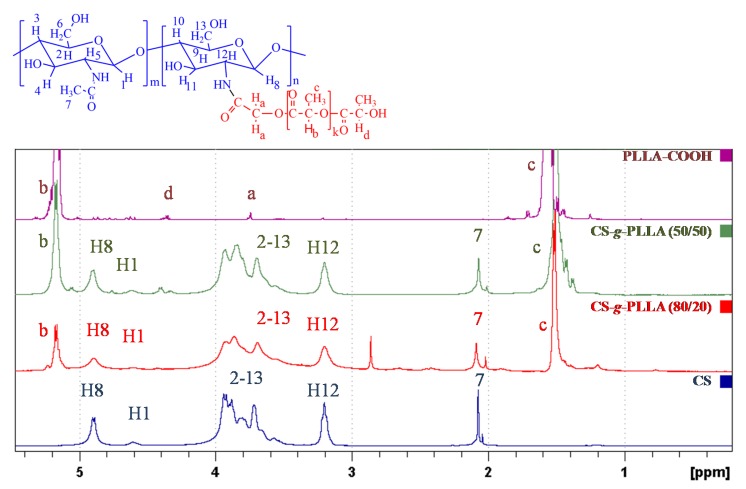
^1^H nuclear magnetic resonance (NMR) spectra of PLLA-COOH in CDCl_3_ and CS-*g*-PLLA(50/50), CS-*g*-PLLA(80/20) and CS in D_2_O:CF_3_COOD(1:1).

PLLA functionalized with one terminal carboxylic acid moiety (PLLA-COOH) was prepared by the ring-opening polymerization of LLA using glycolic acid (GA) as the initiator and an organic base, DMAP [33] as the catalyst (Scheme 1, step a). DMAP was employed instead of the most commonly used organometallic catalyst (stannous octanoate) to avoid metal contamination of the products. The molecular weight and molecular weight distribution of PLLA-COOH were determined by GPC and were found *M*_n_ = 8100 g/mol and *M*_w_/*M*_n_ = 1.26, respectively, while the chemical structure of the PLAA-COOH homopolymer was verified by ^1^H NMR spectroscopy. The ^1^H NMR spectrum of PLLA-COOH showed a characteristic peak at 1.59 ppm and a quartet at 5.16 ppm, corresponding to the methyl (Hc) and methine (Hb) protons of the lactide repeat unit, respectively (Figure 1). The presence of a peak at 3.74 ppm was attributed to the methylene (Ha) protons of the initiator (GA) and verified the presence of the carboxylic acid end-group functionality on the synthesized polymer, while the peak at 4.39 ppm corresponds to the Hd of PLLA. The degree of polymerization (DP) of PLLA-COOH was calculated by rationing the integrals of the peaks attributed to the Hd and Hc protons of the polymer, and was found to be DP = 119, which corresponds to a molecular weight of *M*_n_ = 8600 g/mol, in good agreement with the GPC results, given the difference in the hydrodynamic volume of PLLA-COOH and the PMMA standards used in GPC. 

### 3.2. Synthesis of the Chitosan-g-poly(l-lactide) Copolymers

Two CS-*g*-PLLA copolymers were prepared, CS-*g*-PLLA(80/20) and CS-*g*-PLLA (50/50), containing 82 and 55 wt % CS, respectively. For the synthesis of the graft copolymers a three-step synthetic procedure was followed (see Scheme 1b,c). First, the carboxylic acid terminal group of PLLA-COOH was activated by reaction with DCC and NHS to obtain PLLA-NHS (Scheme 1, Step b). Next, CS was modified by interaction with the anionic surfactant SDS, to form an organo-soluble SDS/CS complex (SCC) (Scheme 1, Step c). Finally, PLLA-NHS was reacted with the amine sites of SCC via an amine coupling reaction (Scheme 1, Step d). To obtain the pure graft copolymers, first, the non-grafted PLLA-COOH chains were eliminated by extensive dialysis against DMSO and next, SDS was removed by precipitation of the polymer in a Tris buffer solution (Scheme 1, step e). 

### 3.3. Characterization of the CS-g-PLLA Graft Copolymers

^1^H NMR and FTIR spectroscopies were employed to characterize the synthesized copolymers. Figure 1 shows the ^1^H NMR spectra of the CS-*g*-PLLA(80/20) and CS-*g*-PLLA(50/50) copolymers and the precursor homopolymers CS and PLLA-COOH. The ^1^H NMR spectrum of CS shows multiple peaks between 3.58–3.94 ppm attributed to H2-H6, H9-H11 and H13 and four peaks at 2.09 ppm (H7), 3.22 ppm (H12), 4.63 ppm (H1) and 4.91 ppm (H8), characteristic of the protons of the sugar moieties [23]. The successful grafting of PLLA onto the CS backbone was confirmed by the presence of the methyl and methine peaks of PLLA, at 1.54 ppm (Hc) and 5.17 ppm (Hb), respectively, in the ^1^H NMR spectra of CS-*g*-PLLA(80/20) and CS-*g*-PLLA(50/50). By ratioing the integrals of the peaks attributed to the (Hc) protons of PLLA at 1.54 ppm, to the methyl protons of the acetylated (H7) CS at 2.09 ppm, the grafting density of PLLA was found to be one PLLA chain per 227 CS repeat units, which corresponds to a 18 wt % PLLA, for CS-*g*-PLLA(80/20), and one PLLA chain per 62 monomer repeat units of CS, corresponding to about 45 wt % PLLA, for CS-*g*-PLLA(50/50). From the above results, it is revealed that for both CS-*g*-PLLA copolymers about 90% of the PLLA chains from the reaction feed were grafted along the CS backbone.

**Figure 2:**
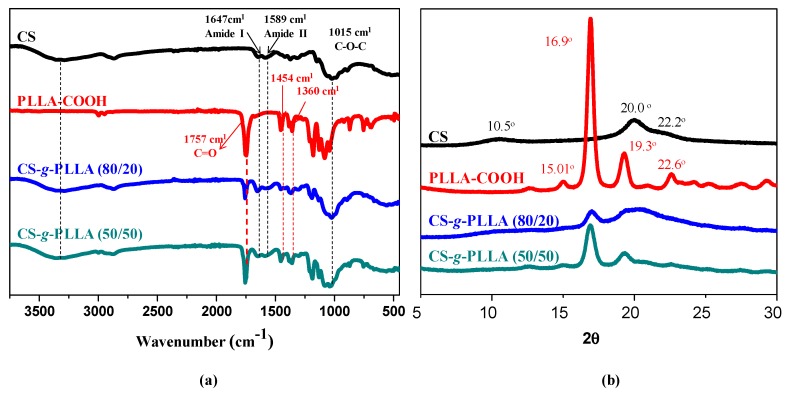
(**a**) Attenuated total reflectance Fourier-transform infrared (ATR-FTIR) spectra and (**b**) X-ray diffraction (XRD) patterns of chitosan (CS) (black line), PLLA-COOH (red line), CS-*g*-PLLA(80/20) (blue line) and CS-*g*-PLLA(50/50) (green line).

The ATR-FTIR spectra for both graft copolymers exhibited the characteristic bands of PLLA and CS verifying the successful grafting of the PLLA chain onto the CS backbone (Figure 2a). In particular, the spectra of CS-*g*-PLLA(80/20) and CS-*g*-PLLA(50/50) showed the carbonyl (C=O) band of the ester group at 1757 cm^−1^, the characteristic C–O–C band at 1015 cm^−1^, the C–H band of the methyl groups at 1454 cm^−1^ and the C-H bending vibration of the methine groups at 1360 cm^−1^ were attributed to the PLLA chains. Moreover, they showed two characteristic peaks at 1647 and 1589 cm^−1^, assigned to the amide I and amide II bonds of CS, respectively [40,41]. An increase in the relative intensity of the carbonyl band at 1757 cm^−1^, due to the PLLA ester groups, compared to the amide I band of CS at 1647 cm^−1^, was observed for CS-*g*-PLLA(50/50), indicating the higher PLLA content of the copolymer.

XRD measurements were performed to further examine the microstructure of the graft copolymers (Figure 2b). The XRD pattern of PLLA-COOH showed four sharp peaks at 15.01°, 16.9°, 19.3° and 22.6° which correspond to the characteristic (010), (110)/(200), (203) and (210) planes of a-phase PLLA [42,43]. The XRD pattern of CS exhibits two characteristic broad peaks at 10.5° and 20.0°, which correspond to the hydrated crystalline structure and to the relative regular crystal lattice (110, 040) of CS, respectively, and a shoulder at 22.2° signifying the presence of an amorphous polymer region [16,44]. The XRD patterns of the CS-*g*-PLLA copolymers showed the two characteristic peaks of PLLA at 16.9° and 19.3°, and a broad peak at 20.0°, assigned to the crystal structure of CS. The latter peak appears broader for the copolymers compared to the respective peak of CS, indicating that the grafting of the PLLA chains along the CS backbone suppresses its crystallinity. The lower intensity of the peak at 20.0° for the CS-*g*-PLLA(50/50) copolymer is attributed to the lower CS content of the copolymer, but could also indicate that the crystallinity of CS has been further decreased, due to the higher PLLA content of the copolymer. Furthermore, the crystallinity index (CrI %) of CS was estimated for pure CS, and for the two graft copolymers using the following equation:

CrI % = ((I_o_ − I_am_)/I_o_) × 100 (2)

where I_o_ is the maximum intensity of the peak at 2θ = 20.0° and I_am_ is the amorphous diffraction signal at 2θ = 16.0° [45,46]. From the above equation, the CrI % of CS was calculated 72% for pure CS, whereas the CrI % of CS upon grafting the PLLA chains was reduced to 62% for CS-*g*-PLLA(80/20) and 29% for CS-*g*-PLLA(50/50). The above results suggest that, by grafting 18 *w*/*w* % PLLA chains along the CS backbone, its crystallinity decreased by 14%, whereas 45 *w*/*w*% PLLA chains reduced the crystallinity of CS by 60%.

### 3.4. Thermal Properties of the CS-g-PLLA Copolymers

**Figure 3:**
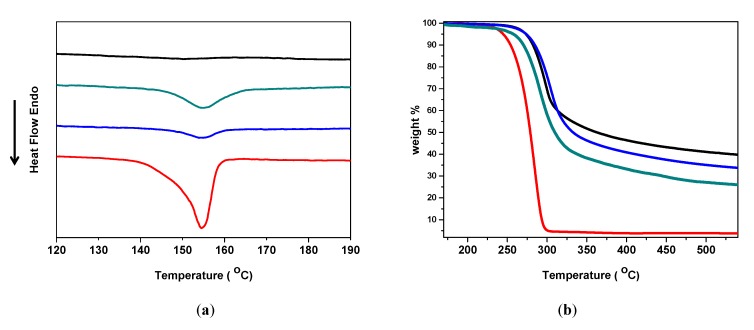
(**a**) Differential scanning calorimetry (DSC) thermographs (first heating cycle) and (**b**) thermogravimetric analysis (TGA) curves for CS (black line), PLLA-COOH (red line), CS-*g*-PLLA(80/20) (blue line) and CS-*g*-PLLA(50/50) (green line).

The thermal properties of PLLA-COOH, CS and the two graft copolymers were investigated by DSC measurements (Figure 3a). The thermogram of the PLLA-COOH homopolymer showed a melting temperature of 154 °C, while CS did not exhibit any transition in the temperature range examined. Upon grafting the PLLA-COOH chains onto the CS backbone, the melting temperature of the PLLA segments remained at 155 °C for both graft copolymers, however, the peak intensity decreased, suggesting that the crystallinity of the PLLA segments was significantly suppressed. The degree of crystallinity of the PLLA-COOH homopolymer and the graft copolymers was calculated from the enthalpy of fusion (Δ*H_f_*). For the PLLA-COOH homopolymer, the Δ*H_f_* was found to be 62.1 J/g, which corresponds to a crystallinity (*X_c_*) of 66% (the Δ*H_f_* for 100% crystalline PLLA is 93.6 J/g) [41]. After grafting the PLLA chains onto the CS backbone, the crystallinity of PLLA reduced to 57 % for CS-*g*-PLLA(50/50) (Δ*H_f_* = 53.8 J/s) and 47% for CS-*g*-PLLA(80/20), (Δ*H_f_* = 44.2 J/s) (Table 1). These results reveal a reduction in the crystallinity of the grafted PLLA chains, which decreases with the decrease in the PLLA content of the copolymer and is in good agreement with the XRD measurements discussed above. 

Thermogravimetric analysis was employed to examine the thermal stability of the graft copolymers and compare it to that of CS and PLLA-COOH (Figure 3b). The TGA curve of CS showed a sharp decrease in mass between 260 and 320 °C, with a maximum decomposition rate at 300 °C and a weight loss of 57%, attributed to the decomposition of CS, mostly via a deacetylation and depolymerization process [47]. At temperatures above 320 °C, a continuous decrease in the sample weight was observed up to 500 °C, at which temperature ~ 41 wt % of the material remained as residue. On the other hand, PLLA-COOH exhibited a lower decomposition temperature with a sharp degradation step (down to 4.3 wt %) at 283 °C. The degradation profiles of the graft copolymers were similar to that of CS. In particular, the copolymer with the lower PLLA content (CS-*g*-PLLA(80/20)) exhibited a sharp degradation step at 303 °C, whereas the graft copolymer with the higher PLLA content (CS-*g*-PLLA(50/50)), was slightly less stable, with a degradation temperature of 291 °C. The decrease in the thermal stability of the graft copolymer as the PLLA content increased was attributed to both the presence of the less thermally stable PLLA, as well as the decrease in the crystallinity of CS [24,28,40]. The absence of a two-step degradation process, attributed to the two homopolymers, in the TGA curves of the copolymers, prohibited the calculation of the copolymer composition by TGA. Furthermore, both CS-*g*-PLLA(80/20) and CS-*g*-PLLA(50/50) showed a continuous drift in their weight up to 500 °C and a ~35% and 27% residual weight, respectively, similar to that found for CS.

### 3.5. Characterization of the Copolymer Films

**Figure 4:**
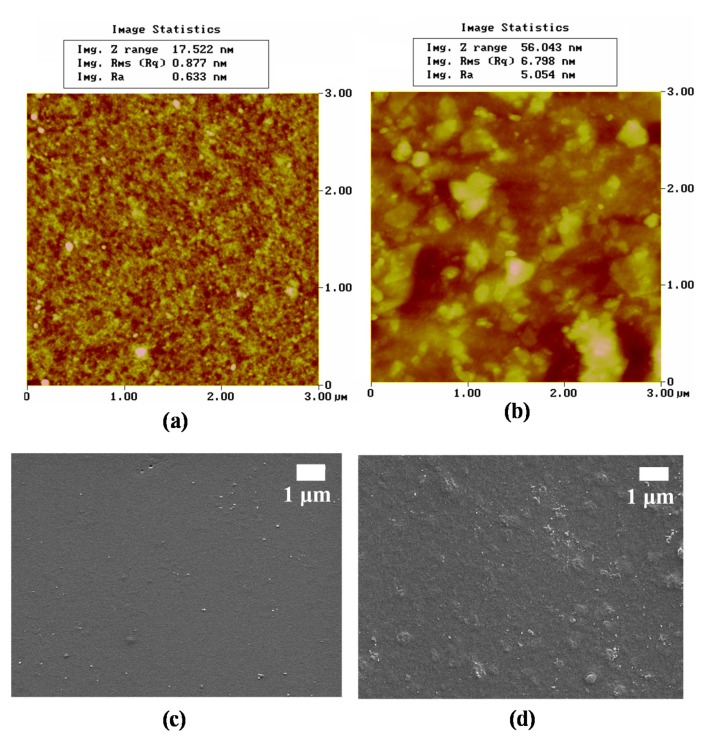
(**a**,**b**) AFM and (**c**,**d**) SEM images of CS-*g*-PLLA(80/20) (**a**,**c**) and CS-*g*-PLLA(50/50) (**b**,**d**).

The surface morphology of the CS-*g*-PLLA copolymer films was investigated by AFM and SEM (Figure 4). AFM analysis of the copolymer films showed a relatively smooth and homogeneous surface for CS-*g*-PLLA(80/20) with a route mean square (RMS) of 0.877 nm (Figure 4a), and a high roughness surface, with a mean RMS of 6.798 nm, for CS-*g*-PLLA(50/50) (Figure 4b). Similar results were found by SEM, which again indicated a smoother surface for the CS-*g*-PLLA(80/20) copolymer films (Figure 4c).

The surface wettability is an important surface characteristic which strongly affects cell adhesion and proliferation. The wettability of the CS-*g*-PLLA films was studied by static water contact angle measurements. A water contact angle of 73° ± 4° and 75° ± 5° was found for the CS-*g*-PLLA(80/20) and CS-*g*-PLLA(50/50) films, respectively, indicating a moderately hydrophilic surface for both copolymer films. 

### 3.6. Nanoindentation on the CS-g-PLLA Discs in the Dry and Wet State

**Figure 5:**
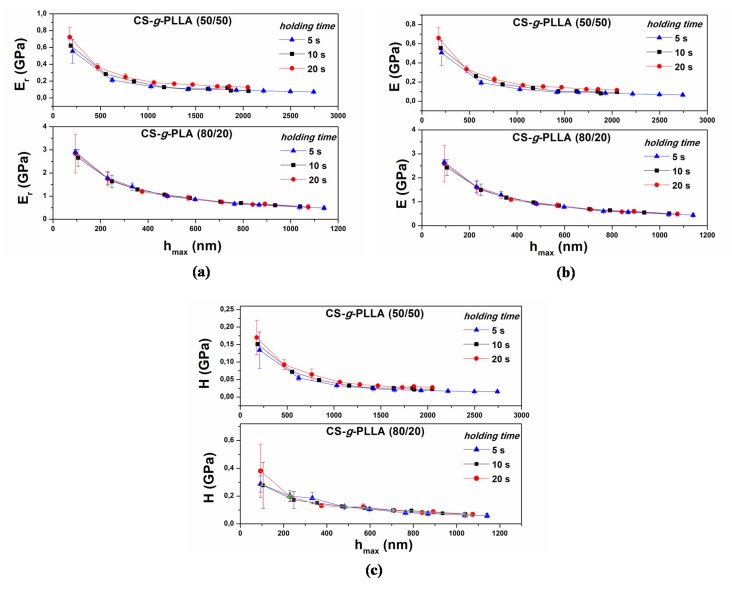
(**a**) Reduced modulus (Er), (**b**) Young’s modulus (E) and (**c**) hardness (H) values for the CS-*g*-PLLA copolymers as a function of maximum indentation depth, for 5, 10 and 20 s holding times.

Nanoindentation tests were performed, at various applied loads and holding times, reaching different maximum penetration depths, on the CS-*g*-PLLA discs. Figure 5 presents the reduced modulus (Er), Young’s modulus (E) and hardness (H) values for 5, 10 and 20 s holding times, respectively, following the load control mode for both CS-*g*-PLLA copolymers. In all cases, the H, E and Er values are higher at low penetration depths, whereas, as the indenter penetrated further into the sample, the values decreased and finally reached a plateau at a minimum value. The CS-*g*-PLLA(80/20) copolymer with the higher CS content showed higher Er, E and H values compared to CS-*g*-PLLA(50/50), which was attributed to the formation of more interchain hydrogen bonds between the amino and hydroxyl functionalities of CS (Table 2) [48]. In general, it was found that the graft copolymer with the higher PLLA content presented inferior nanomechanical properties compared to that with the higher CS content.

The CS-*g*-PLLA(80/20) sample presented the higher resistance to applied load and possessed the higher Er, E and H values, whereas the graft copolymer with the higher PLLA content presented lower nanomechanical properties, as discussed previously by Y. Wan et al. [49]. CS is a semi-crystalline polymer and, after being grafted with PLLA side chains, the crystallization of CS is significantly reduced from 72% to 62% and 29% for CS-g-PLLA(80/20) and CS-g-PLLA(50/50), respectively determined by XRD analysis, which results in poorer mechanical properties.

**Figure 6:**
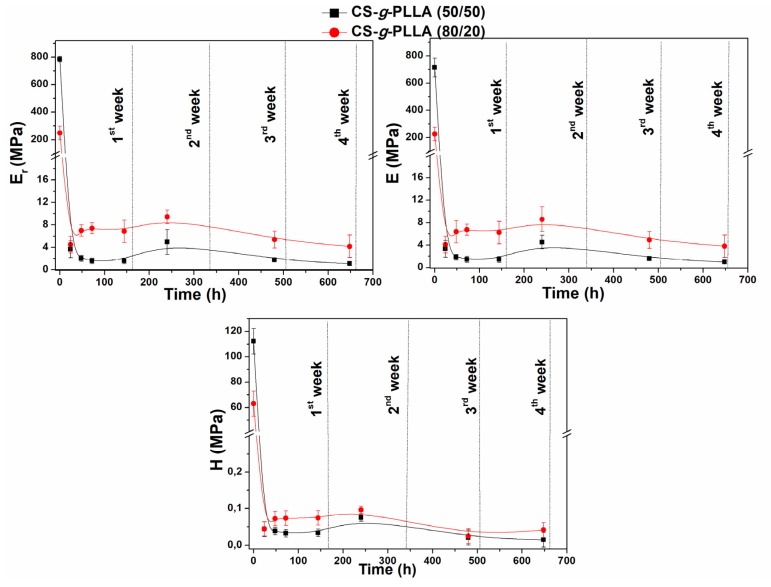
Reduced modulus (Er), Young’s modulus (E) and hardness (H) values for the CS-*g*-PLLA copolymers at 500 μΝ as a function of immersion time in α-MEM medium.

Following the nanoindentation tests performed on the dried discs, the samples were immersed in α-MEM medium at 25 °C under static conditions, for a period of 4 weeks, and their nanomechanical properties were evaluated at certain time periods. Due to the hydrophilic nature of CS, the copolymer with the higher CS content, was highly deformed after the 4 week immersion in the medium, whereas the CS-*g*-PLLA(50/50) sample became swollen and fractured for the same time period. Figure 6 shows the reduced modulus, Young’s modulus and hardness values, calculated by the Oliver and Pharr method, for both CS-*g*-PLLA samples as a function of immersion time. As seen in the figure, for both CS-*g*-PLLA samples, the Er, E and H values decreased rapidly during the first 24 h of submersion in the medium, due to the water uptake by the samples (Table 3). 

The kinetics of the scission of the ester bonds of the aliphatic polyesters, which control their hydrolytic rate, are higher in the amorphous regions, in which the water molecules can penetrate faster within the disordered polymer, compared to the crystalline domains, and therefore increases with the decrease in polymer crystallinity [50,51,52]. As a result, the fast degradation rate of the CS-*g*-PLLA discs is attributed to the swelling of the material due to the presence of the hydrophilic CS component and the lower crystallinity of the polymer chains in the graft copolymer. However, despite the softening and the high degradation rate of the CS-*g*-PLLA copolymers, the samples presented adequate mechanical integrity under the applied load at the fully hydrated state for a period of 4 weeks.

### 3.7. Degradation Profile of the CS-g-PLLA Discs

**Figure 7:**
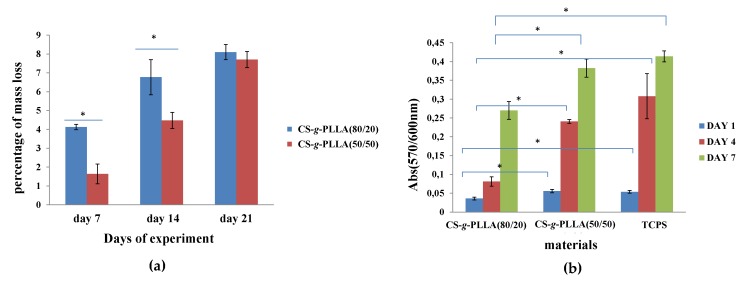
(**a**) Percentage of mass loss for the two graft copolymers, CS-*g*-PLLA(80/20) and CS-*g*-PLLA(50/50), following immersion in PBS buffer at 37 °C for 7, 14 and 21 days; (**b**) Cell metabolic activity of pre-osteoblasts cultured on CS-*g*-PLLA(80/20) and CS-*g*-PLLA(50/50)) for 1, 4 and 7 days. TCPS was used as a control for the same timepoints. Each bar represents the mean ± standard error of the mean of triplicate wells from two independent experiments.

The percentage of mass loss of the two graft copolymers, CS-g-PLLA(80/20) and CS-*g*-PLLA(50/50), following immersion of the copolymer discs in PBS for 7, 14 and 21 days, was determined (Figure 7a). The weight loss was higher for the graft copolymer with the higher CS content for the first two weeks. Specifically, the mass loss for the CS-*g*-PLLA(80/20) discs was ~ 4, 7 and 8 wt % at days 7, 14 and 21, respectively, whereas the corresponding values for the CS-*g*-PLLA(50/50) sample were ~2, 4 and 8 wt %. A similar behavior has been reported in the literature, and was attributed to the higher content of the material in hydrophilic CS, which provided better accessibility to water, thus enhancing its hydrolytic degradation [53]. 

### 3.8. Viability and Proliferation of Pre-osteoblastic Cells on the CS-g-PLLA Films

The adhesion and proliferation of the cells on the biomaterial surface is a key step in tissue engineering. The adhesion and growth of MC3T3-E1 cells on the CS-*g*-PLLA films were determined by the colorimetric PrestoBlue^®^ assay. A comparison of the cellular metabolic activity on the two copolymer films after 1, 4 and 7 days in culture is depicted in Figure 7b, which shows that the number of viable cells was significantly higher on the CS-*g*-PLLA(50/50) films for all timepoints. In all samples, a few pre-osteoblastic cells adhere and grow in Day 1. For CS-*g*-PLLA(80/20), the cell proliferation increased significantly from Day 1 to 4 and up to Day 7. Similarly for CS-*g*-PLLA(50/50), the cell proliferation increased from Day 1 to 4 and up to Day 7, demonstrating a significant rise at all three timepoints compared to the CS-*g*-PLLA(80/20) counterpart. The viability of the cells cultured on CS-*g*-PLLA films was similar to TCPS, used as a reference surface. The above results suggest that the CS-*g*-PLLA copolymer with the higher PLLA content (CS-*g*-PLLA(50/50)) promotes pre-osteoblastic cell adhesion, viability and proliferation, and is thus a promising biomaterial for bone tissue engineering.

### 3.9. Morphology of ΜC3T3-E1 Cells on the CS-g-PLLA Film

**Figure 8:**
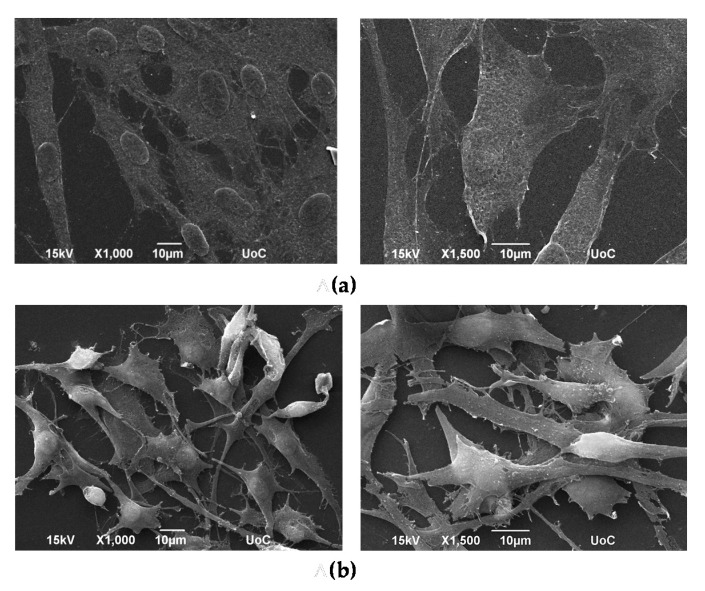
Characteristic SEM images illustrating the morphology of the MC3T3-E1 pre-osteoblastic cells cultured for 2 days on (**a**) CS-*g*-PLLA(80/20) and (**b**) CS-*g*-PLLA(50/50) films.

The pre-osteoblastic cell morphology on the CS-*g*-PLLA(80/20) and CS-*g*-PLLA(50/50) films was investigated by SEM. Figure 8 shows the morphology of the cells cultured on the two copolymer films for 2 days. A high number of cells adhered on both biomaterial surfaces. The cells exhibited distinct morphological characteristics and retained their typical fibroblast-like polygonal morphology with long spindle-like cellular extensions and good spreading on the copolymer surfaces. Furthermore, the number of ΜC3T3-E1 cells on the CS-*g*-PLLA(50/50) films was higher than on the CS-*g*-PLLA(80/20) films, which is in good agreement with the viability experiments presented above. These results indicate that both graft copolymers promote cell attachment on the biomaterial surfaces.

## 4. Discussion

Biomaterials for scaffold fabrication are one of the main components in tissue engineering. CS and PLLA have been extensively employed in bone tissue engineering. The combination of CS and PLLA, which possess complementary advantageous properties, provides an ideal scaffold system with multifunctional properties. To date, these materials have been combined either in the form of blends to prepare porous scaffolds [15,54], or as PLLA fibers [18] and films [19] surface-modified with CS. 

In vitro experiments have shown that the combination of CS with PLLA is favorable for the attachment and proliferation of different cell types. For example, a core-shell or island-like electrospun scaffold, in which CS was deposited on the surface of PLLA fibers, has been shown to be beneficial for the attachment and growth of MC3T3-E1 cells compared to the two polymers alone [55]. Moreover, 3D-printed macroporous PLLA scaffolds, surface-modified with CS, showed improved cell viability for human mesenchymal stem cells (hMSCs) compared with a PLLA construct [20]. In another report, mechanical and in vitro studies on CS/PLLA scaffolds prepared by an emulsion freeze-drying process, with a CS/PLLA weight ratio of 2/1, 2/2 and 2/3, showed that the compressive modulus and the compressive strength of the scaffolds increased with the PLLA content, while the scaffold with a 2/2 CS/PLLA weight ratio presented comparable MC3T3-E1 cell viability to the CS scaffold, much higher than that of PLLA alone [15]. However, only a few studies have examined the potential of CS-*g*-PLLA copolymers as biomaterials for tissue engineering [25,32]. Recent reports from our research group focused on the development of CS grafted with polycaprolactone chains, demonstrating their potential in hard and soft tissue engineering, as they support the proliferation of pre-osteoblastic cells [56], Wharton’s jelly mesenchymal stem cells [35,52], and macrophages, acting as an immunoregulator for their polarization [57].

In this work, the two biomaterials CS and PLLA were combined by grafting PLLA chains onto the CS backbone to obtain CS-*g*-PLLA copolymers, and the response of pre-osteoblastic cells on the hybrid biomaterials was examined for the first time. The advantage of this approach is that CS is not coated on the surface of the material, but is homogeneously distributed within the biomaterial at the nanometer size range. Previously, in vitro experiments on CS-*g*-PLLA electrospun fibers, prepared by the “grafting from” technique at a 1/4 to 1/24 CS/PLLA mole ratio, showed that the viability of fibroblast L929 cells increased with the PLLA content of the copolymer, and was higher than that of CS and comparable to that of PLLA [25]. In another study, Wan et al. developed CS-*g*-PLA electrospun fibers at a 2/1 to 1/4 CS/LA weight ratio and showed that, by raising the PLA content, the Young’s modulus of the fibers increased, while the viability of fibroblast cells on the CS-*g*-PLA fibers was similar to that of CS and was not significantly affected by the PLA content of the copolymer [31]. Furthermore, CS-*g*-PLLA 3D scaffolds with different PLLA contents were developed by the “*grafting from*” approach by varying the CS/LLA weight ratio in the feed from 1/2 to 1/4. The growth rate of BMSCs on the CS-*g*-PLLA copolymer surface was higher than that of CS and PLLA alone, and increased as the CS/LLA feed ratio decreased for the first days in culture, however, after the third day, the copolymer with the lower PLLA content favored the growth of the cells [30].

In the present work, we examined the effect of the PLLA content of the copolymer on the growth and viability of MC3T3-E1 cells using two CS-*g*-PLLA graft copolymers of different compositions, CS-*g*-PLLA(80/20) and CS-g-PLLA(50/50), with a CS content of 82 wt % and 55 wt %, respectively. The in vitro evaluation of pre-osteoblastic cells showed that both CS-g-PLLA graft copolymers support the attachment of MC3T3-E1 cells on their surface. Furthermore, viability experiments showed that the number of alive cells was higher on the graft copolymer with the higher PLLA content, CS-*g*-PLLA(50/50), from Day 1 up to Day 14, which is in good agreement with the results obtained by SEM. The behavior of the MC3T3-E1 cells can be correlated with the lower degradation rate of CS-g-PLLA(50/50) because the degradation of PLLA leads to the formation of lactic acid, which reduces the pH of the culture medium and adversely affects the viability of the MC3T3-E1 cells [25]. To conclude, both CS-*g*-PLLA copolymers promoted MC3T3-E1 cell adhesion and proliferation. However, the copolymer with the higher PLLA content favored the viability and growth of the cells, rendering it a promising biomaterial for bone tissue engineering applications.

## 5. Conclusions

In this study, CS-*g*-PLLA copolymers of two different compositions, CS-*g*-PLLA(80/20) and CS-*g*-PLLA(50/50), with a CS content of 82 wt % and 55 wt %, respectively, were prepared. The chemical structure and copolymer composition were investigated by ^1^H NMR and FTIR spectroscopies, which verified the successful synthesis of the graft copolymers. The thermal stability of the two copolymers was examined by TGA and the degradation temperature was found to decrease with the CS content of the copolymer, from 303 °C for CS-*g*-PLLA(80/20) to 291 °C for CS-*g*-PLLA(50/50). DSC studies showed that the crystallinity of PLLA decreased from 57% to 47% with the increase in the CS content of the graft copolymer. This resulted in lower hardness and Young modulus values for the CS-*g*-PLLA(80/20) copolymer following immersion in α-MEM medium, however both samples retained sufficient integrity under mechanical stress after 4 weeks degradation in the cell culture medium at 25 °C, suggesting that they are promising candidates for use in tissue engineering. Finally, the copolymer films were used to investigate the adhesion and growth of pre-osteoblastic MC3T3-E1 cells. The cells adhered strongly and grew on both materials, whereas the copolymer with the higher PLLA content promoted a higher proliferation of pre-osteoblastic cells. The above results reveal the potential of both graft copolymers, and in particular of CS-*g*-PLLA(50/50), for use in scaffold fabrication for bone tissue engineering applications. 

## Figures and Tables

**Table 1:** Thermal properties of the CS-*g*-PLLA copolymers.

Sample	T_m_ (°C)	ΔH_f_ (J/g) ^a^	Χ_c_
PLLA-COOH	154	62.1	66%
CS-*g*-PLLA(80/20)	155	44.2	47%
CS-*g*-PLLA(50/50)	155	53.8	57%

^a^ enthalpy of fusion.

**Table 2:** Reduced modulus (Er), Young’s modulus (E) and hardness (H) values of the CS-*g*-PLLA copolymers at 1000 nm indentation depth following 5 s holding time and 10 s loading and unloading time.

Sample	Er (GPa)	E (GPa)	H (GPa)
**CS-*g*-PLLA(80/20)**	0.53 ± 0.11	0.48 ± 0.01	0.06 ± 0.02
**CS-*g*-PLLA(50/50)**	0.14 ± 0.01	0.13 ± 0.02	0.03 ± 0.01

**Table 3:** Reduced modulus (Er), Young’s modulus (E) and hardness (H) values at 500 nm indentation depth following immersion in α-MEM for different time periods.

Time (h)	Er (MPa)		E (MPa)		H (MPa)	
	CS-*g*-PLLA (80/20)	CS-*g*-PLLA (50/50)	CS-*g*-PLLA (80/20)	CS-*g*-PLLA (50/50)	CS-*g*-PLLA (80/20)	CS-*g*-PLLA (50/50)
**0**	785 ± 20	250 ± 50	715 ± 70	226 ± 50	112 ± 10	63 ± 0
**24**	3.7 ± 2	4.5 ± 1.5	3.3 ± 1.5	4.1 ± 1.5	0.04 ± 0.02	0.04 ± 0.02
**648**	1.1 ± 0.4	4.2 ± 2	1.0 ± 0.2	3.8 ± 2	0.01 ± 0.01	0.04 ± 0.02
